# Pilot study on comparisons between the effectiveness of mobile video-guided and paper-based home exercise programs on improving exercise adherence, self-efficacy for exercise and functional outcomes of patients with stroke with 3-month follow-up: A single-blind randomized controlled trial

**DOI:** 10.1142/S1013702520500079

**Published:** 2020-02-20

**Authors:** Bryan Ping Ho Chung, Wendy Kam Ha Chiang, Herman Lau, Titanic Fuk On Lau, Charles Wai Kin Lai, Claudia Sin Yi Sit, Ka Yan Chan, Chau Yee Yeung, Tak Man Lo, Elsie Hui, Jenny Shun Wah Lee

**Affiliations:** 1 Physiotherapy Department, Tai Po Hospital, 11 Chuen On Road, Tai Po, New Territories, Hong Kong; 2 Physiotherapy Department, Shatin Hospital, 33A Kung Kok Street, Shatin, New Territories, Hong Kong; 3 Hospital Chief Executive, Shatin Hospital, 33A Kung Kok Street, Shatin, New Territories, Hong Kong; 4 Department of Medicine and Geriatrics, Shatin Hospital, 33A Kung Kok Street, Shatin, New Territories, Hong Kong; 5 Department of Medicine and Geriatrics, Tai Po Hospital, 11 Chuen On Road, Tai Po, New Territories, Hong Kong; taipobryan@yahoo.com

**Keywords:** Physiotherapy, stroke, rehabilitation, exercise, adherence, self-efficacy, functional outcome, video, home

## Abstract

**Objective:**

To compare the effectiveness of mobile video-guided home exercise program and standard paper-based home exercise program.

**Methods:**

Eligible participants were randomly assigned to either experimental group with mobile video-guided home exercise program or control group with home exercise program in a standard pamphlet for three months. The primary outcome was exercise adherence. The secondary outcomes were self-efficacy for exercise by Self-Efficacy for Exercise (SEE) Scale; and functional outcomes including mobility level by Modified Functional Ambulatory Category (MFAC) and basic activities of daily living (ADL) by Modified Barthel Index (MBI). All outcomes were captured by phone interviews at 1 day, 1 month and 3 months after the participants were discharged from the hospitals.

**Results:**

A total of 56 participants were allocated to the experimental group (n=27) and control group (n=29). There were a significant between-group differences in 3-months exercise adherence (experimental group: 75.6%; control group: 55.2%); significant between-group differences in 1-month SEE (experimental group: 58.4; control group: 43.3) and 3-month SEE (experimental group: 62.2; control group: 45.6). For functional outcomes, there were significant between-group differences in 3-month MFAC gain (experimental group: 1.7; control group: 1.0). There were no between-group differences in MBI gain.

**Conclusion:**

The use of mobile video-guided home exercise program was superior to standard paper-based home exercise program in exercise adherence, SEE and mobility gain but not basic ADL gain for patients recovering from stroke.

## Introduction

Stroke, also known as cerebrovascular accident, is an acute disturbance of focal or global cerebral function with signs and symptoms lasting more than 24 h or leading to death presumably of vascular origin.^1^ It is the third leading cause of death in Hong Kong after cancer and heart disease. More than 3000 people died in Hong Kong each year for this condition.^2^ The most widely recognized impairment caused by stroke is motor impairment, which restricts function in muscle movement or mobility.^3,4^ It was shown that patients with stroke who had as much practice as possible within 6 months after stroke onset could achieve the best functional outcome.^5-7^

In Hong Kong, under the service model of Hospital Authority, patients would be referred to ambulatory services, such as geriatric day hospital, or domestic physiotherapy services to continue their stroke rehabilitation after discharge from hospital. However, some patients could not attend ambulatory services due to various difficulties, such as transportation and absence of carers. The low frequency of domestic physiotherapy service also reduced the effectiveness of rehabilitation of patients. From our local data, around a quarter of home-care patients have not received any further rehabilitation training from ambulatory or domestic physiotherapy services. Among this population, there were approximately 56% of them rated to be Modified Functional Ambulatory Category (MFAC) 2–5, who may have had higher potential of improvement in stroke outcomes if appropriate rehabilitation training was given.^8^ Home-based exercise program would be a good choice for filling this post-discharge gap of rehabilitation continuation.

Traditionally, for continuation of exercise training program, physiotherapists would prescribe home exercises programs in paper-based format.^9,10^ However, evidence showed that home exercise prescription in paper-based format does not lead to better adherence to a home exercise program compared to having no written and pictorial instructions for patients with stroke less than four months.^10^

The recent increasing accessibility of smart technology^11^ offers an opportunity to advance the mode of delivery of home exercise program by mobile devices such as video-guided exercise on electronic tablets^12^ and video-guided exercise on mobile apps.^13^ However, the effects of mobile video-guided exercise programs were controversial. A study showed that home exercise programs filmed on an electronic tablet, with an automated reminder, were not superior to standard paper-based home exercise programs in terms of adherence, motor function, or satisfaction for patients recovering from stroke.^12^ In contrast, another study showed that people with musculoskeletal conditions who adhere better to their home exercise programs in video-based format are provided with an app with remote support compared to paper handouts; although the clinical importance of this added adherence is unclear.^13^

Since one of the major outcomes to evaluate the effectiveness of the mode of delivery of home exercise program is adherence,^10,12,13^ it is worthwhile to study the factor affecting adherence of mode of delivery of home exercise program. It was suggested that one of the barriers to adherence of home exercise is low self-efficacy.^14,15^ According to the theory of self-efficacy, self-efficacy is defined as a person’s confidence in their ability to perform a task.^16^ Thus, self-efficacy plays an important role in maintaining the exercises behavior after stroke^17-19^ and improving self-efficacy for exercise (SEE) could influence long-term exercise behavior as well as the early stages of exercise adoption.^20-23^ Another factor affecting adherence of home exercise was the delivery mode of training program.^13^ Studies showed that visual information brings more benefits to patients than verbal information alone.^24-27^ Adherence of home exercise program has positive correlation with physical function and physical performance of patients.^15,28^

It is worthwhile for physiotherapists to investigate the mode of delivery to enhance exercise adherence and self-efficacy of post-discharge home exercise for patients with stroke. The objective of the study is to compare the effectiveness of video-guided exercise program and standard paper-based home exercise program on adherence of exercise, self-efficacy and functional outcomes in patients with stroke within 3-month follow-up.

## Methods

A randomized, controlled, assessor-blinded clinical trial was conducted between July 2018 and June 2019. Participants were recruited from the inpatient Stroke Rehabilitation Program in the Department of Medicine and Geriatrics of Tai Po Hospital and Shatin Hospital. The two hospitals provided multidisciplinary inpatient stroke rehabilitation to more than 1000 stroke patients yearly. All those stroke patients were diagnosed with acute stroke and were transferred from all the three acute hospitals in New Territories East Cluster. The hypothesis was that participants prescribed with video-guided home exercise will demonstrate higher adherence of exercise, better self-efficacy and better functional outcomes when compared with the participants in paper-based exercise prescription group.

Participants were eligible for inclusion if their principle diagnosis was stroke, participants or their carers have smart devices such as smart phones or tablets that are able to scan QR code and connect to the Internet as well. All the participants or carers could read Chinese. They were excluded if (i) could not follow gesture and instructions; (ii) MFAC upon discharge was below 2 or above 5, (iii) no smart device for video and (iv) refusal.

This was a single-blinded randomized study. The study procedure flowchart is presented in Appendix B. Before enrolment and randomization, potential participants were screened by their case therapists to ensure they met the inclusion criteria and none of the exclusion criteria. Eligible participants were then randomly assigned to either Intervention Group or Control Group in a 1:1 ratio. Investigators who were responsible for data collection were blinded to the group allocation. A randomization list was developed by personnel who were not involved in this study and who would not have any contact with the study participants. The details of the list were unknown to any of the investigators and study coordinators, and were contained in a set of sealed, sequentially numbered envelopes. Each enrolled participant was allocated to the next sequential number on the list. All research study personnel, including those who collect data and assess outcomes, were blinded to the group assignment. The envelopes and the randomization list were not revealed to any of the study personnel until completion of recruitment and data collection.

Research team adopted per-protocol analysis method and provided two brief sessions to all physiotherapists of the stroke units to inform the process of subject recruitment of potential participants. Case physiotherapists would inform research team for subject recruitment once potential participants have discharge plan, usually 1–2 weeks before discharged. Case physiotherapists provided brief information about the study to potential participants or their carers before their discharge from hospitals. Research team approached the participants and gave detailed information about the study and verifies their interest in participating once verbal consent was obtained. The Joint Chinese University of Hong Kong — New Territories East Cluster Clinical Research Ethics Committee (The Joint CUHK-NTEC CREC) approval was obtained prior to the commencement of the study. This trial design was registered prospectively with the ClinicalTrials.gov Protocol Registration and Results System (ClinicalTrials.gov ID: NCT03509363). Written informed consent was obtained from all participants.

Pre-discharge training sessions lasting for 10–15 min were provided to participants, and their carers if any, of both groups in order to make them familiar with the selected home exercises and the technique of using mobile phone to scan QR for intervention group. Participants allocated to the intervention group were prescribed a set of exercise video with QR code provided in home exercise pamphlets and they had to perform the prescribed exercises under the guidance of the videos. On the other hand, participants in control group were given instructions for their home exercise program in a traditional pamphlet includes photographs and instructions of exercise demonstration. The content of home exercise program in both groups was the same and was based on the recommendations from the National Stroke Foundation Clinical Guidelines, included nine arm control exercises, six leg control exercises, six truck control exercises and four mobility exercises (Table 1).

**Table 1.** Type of home exercises in both experimental group and control group.

Arm control exercise
1. Shoulder elevation in lying (Active) 2. Shoulder elevation in lying (Active-assisted) 3. Elbow flexion/extension (Active) 4. Elbow flexion/extension (Active-assisted) 5. Shoulder elevation in sitting (Active) 6. Shoulder elevation in sitting (Active Assisted) 7. Weight-bearing to affected arm (Active) 8. Shoulder horizontal abduction/adduction (Active) 9. Hand grasp and release (Active)
Leg control exercise
1. Hip abduction/adduction in lying (Both leg) (Active) 2. Hip abduction/adduction in lying (Affected leg) (Active) 3. Hip abduction/adduction in lying (Both leg) (Active-assisted) 4. Hip and knee flexion/extension in lying (Active) 5. Hip and knee flexion/extension in lying (Active-assisted) 6. Knee extension in sitting (Active)
Trunk control exercise
1. Trunk rotation (Active) 2. Trunk rotation (Active-assisted) 3. Double-leg bridging (Active) 4. Single-leg bridging (Active) 5. Double-leg bridging (Active-assisted) 6. Forward reaching (Active)
Mobility
1. Sit to stand (Active Assisted) 2. Weight-shifting in sideway (Active Assisted) 3. Stepping back and forward (Active Assisted) 4. Semi-squat (Active Assisted)

The doses for the participants were prescribed by their case physiotherapists according to the needs and abilities of participants. The total number of home exercise varied from 3 to 5 and the treatment frequencies of each exercise varied from daily to 3 times per day so the course length varied from 10 to 30 min daily. Participants’ needs and the abilities were defined as amount of assistance and the mobility by participants’ pre-discharge MFAC.

All participants were screened by their case physiotherapists according to exercise prescription guidelines of American College of Sports Medicine^29^ for any contraindication for exercise. Only patients who were medically fit for exercise were recruited. Exercise-related adverse events such as chest tightness or pain, dizziness, and/or trip, stumbles, or falls might happen during exercise but which was not as higher risk than the usual prescription of home exercises. Suitability of participating home exercise program will be assessed by physiotherapists based on environmental risk, fall risk, and competence of participants or carers in performing exercise with participants.

All participants’ data related to the research were collected in data collection sheets by case therapists of the patients. The data in the data collection sheets were transferred to a database in Excel format that could be accessed only by the research team members. Therefore, we could judge which participants were included in the analysis. Data on patients’ demographics, gender, age, site of lesion, side of stroke, type of stroke, education level, experience of using mobile device, present of complication such as neglect and dysphasia, cardiovascular risk factors and availability of carer, were retrieved from Central Management System of Hospital Authority by case therapists of the patients.

Outcome measures including self-reported exercise adherence, self-efficacy for exercise (SEE-C), MFAC, and Chinese version of the Modified Barthel Index (MBI) were assessed on phone follow-up basis by a blinded-assessor at 1 day, 1 month, and 3 months after the participants were discharged from hospitals. For between group comparisons, since participants had difference baseline functional status, baseline functional status affects MBI and MFAC but not SEE and Adherence, therefore MBI gain and MFAC gain were used to compare the improvement of functional status for fair comparisons. In addition, since we directly compared Adherence and SEE that 3 time points were tested; while we compared MBI gain and MFAC gain that only 2 time points were tested i.e., 1-month gain (1-month minus baseline) and 3-month gain (3-month minus baseline).

### Self-reported exercise adherence

Since there has no well-developed measures that capture self-reported adherence to prescribed but unsupervised home-based rehabilitation exercises; and since we interviewed the participants by phone, some details could not be recalled. So we adopt the concept visual analogue scale to ask the adherence by percentage (0–100%) as a total effect of adherence of number of session, daily frequent, repetition, set and quality of movement of the whole review period. The exercise adherence was measured by asking participants to report their percentage of exercise completion between 3 period of time i.e., from discharge to 1 day post-discharge, from 1 day post-discharge and 1-month post-discharge and from 1-month post-discharge to 3-month post-discharge during phone follow-up. Log sheets were not used since it was reported that log sheet that needs to be filled out regarding the completion of each exercise would serve as a reminder and a motivational track record for the patient and assists patients in improving their adherence.^31^

### Chinese version of self-efficacy for exercise (SEE-C)

An original English version of the SEE scale was designed to test people’s confidence to continue exercising in the face of barriers to exercise.^32^ This scale has a range of total scores from 0 to 90. A higher score indicates higher SEE. Estimates of the reliability and validity of the nine item SEE scale have been widely tested and shown to be valid for use in various settings, with internal consistency (alpha=0.93) and validity with efficacy expectations significantly related to exercise activity, and factor loadings all greater than 0.50.^33-35^ The Cronbach’s alpha coefficient of Chinese version of the SEE scale was 0.75^35^ and Pearson’s correlation revealed a statistically significant correlation between perceived health and SEE-C score (r=−0.17, p=0.019).^35^

### Modified functional ambulatory category (MFAC)

The MFAC was a 7-point Likert Scale (1–7) that was used to classify a patient’s walking capacity. Gait was divided into seven categories, ranging from no ability to walk and requires manual assistance to sit or is unable to sit for 1 minute without back or hand support (MFAC 1) to the ability to walk independently on level and non-level surfaces, stairs, and inclines (MFAC 7).^36^ The inter-rater reliability of the MFAC (intraclass coefficient [ICC]) was 0.982 (0.971–0.989), with a kappa coefficient of 0.923 and a consistency ratio of 94% for stroke patient^37^ and the ICC of the MFAC in patients with hip fractures is 0.96, with a construct validity of r=0.81 on the Elderly Mobility Scale (EMS).^36^ Participants will be asked to describe their current mobility status via phone follow-up.

### Chinese version of the modified barthel index (MBI-C)

MBI was used to assess patients’ basic activities of daily living (ADL) in this study. MBI measures the participant’s performance on 10 functional items including self-care, continence, and locomotion. The values assigned to each item was based on the amount of physical assistance required to perform the task and added to give a total score ranging from 0 to 100 (0=fully dependent, 100=fully independent) with higher score indicating higher levels of physical function.^38^ There was no subtotal score because there was no subscale.^38^ The internal consistency reliability coefficient for MBI was 0.90.^38^ The Chinese version of MBI has been found to have good validity and reliability for assessing stroke patients.^39^ A study showed that the correlations between the performance-based ADL and the interview-based ADL were r greater than 0.97 for the total score and r greater than 0.85 for most of the individual items.^40^

Although the randomization design of the study reduced the biases which could compromise the outcomes, we analyzed the confounding variables such as age, gender, type of stroke, side of hemiplegia, present of complication such as neglect and dysphasia,^41^ follow-up physiotherapy such as ambulatory day hospital and domiciliary physiotherapy^42^ availability of carer,^43^ and experience of smart device^44^ to indicate any significant association in the study.

Pair t-test and Chi-Square test were used to analysis the between-groups differences of baseline characteristics. Chi-Square test was used to analysis the between-groups of exercise adherence at all three time points i.e., baseline, 1-month, and 3-months. Independent t-test was used to analysis the between-groups of SEE, MFAC gain, and MBI gain at all three time points i.e., baseline, 1-month, and 3-months. Pair t-test was used to analysis the within-group differences between baseline to 1-month and baseline to 3-months of MFAC and MBI. The significance level was set at 0.05 for between-group analysis. All of these analyses were performed with SPSS version 18.0. Those participants with missing data due to loss of contact in phone interviews in three consecutive working days were omitted.

## Results

**Fig. 1.**
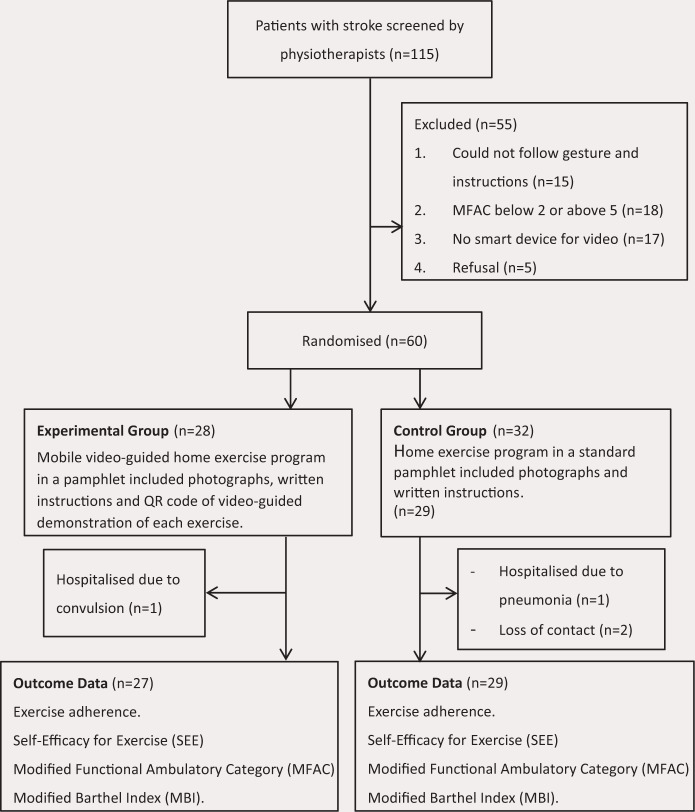
Design and flow of participants through the trial.

There was a total of 115 stroke patients who were screened by physiotherapists for this study from August 2018 to March 2019. Of these, 55 participants were excluded, in which, 15 could not follow gesture and instruction; 18 had mobility below MFAC 5 or above MFAC 5; 17 had no smart device for video watching and 5 refused. The remaining 60 participants were recruited; with 28 randomly allocated to the experience group and 32 randomly allocated to the control group (Fig. 1). There were no adverse events recorded as a result of participation in this trial. Of the 60 participants, there were four withdrawals. One hospitalized due to convulsion, one hospitalized due to pneumonia and two were lost of contact. A total of 56 participants completed the study without missing data.

The groups appeared well matched and no between-group difference in age, gender, time since stroke, type of stroke, side of hemiplegia, complication, site of lesion, follow-up physiotherapy, type of carer, experience in smart device, baseline SEE and baseline MFAC but slight difference in baseline MBI. Mean age of participants was 69.8 years (SD 14.9). The mean time since stroke was 39.5 (SD 15.3) (Table 2).

**Table 2.** Baseline characteristics of participants.

Characteristic	Total (n=56)	Exp (n=27)	Con (n=29)	T-tests p	Chi-Square p
Participants Age (years), mean (SD), (Range)	69.8(14.9), 38–93	66.9(14.0), 40–93	72.5(15.5), 38–93	0.166	
Gender, n males (%)	31(55.4)	14(51.9)	17(58.6)		0.611
Time since stroke (days), mean (SD), (Range)	39.5(15.3), 11–89	41.3(16.5), 23–89	37.9(14.2), 11–64	0.407	
Type of stroke (%)					0.838
Infract	45(80.4)	22(81.5)	23(79.3)		
Hemorrhage	11(19.6)	5(18.5)	6(20.7)		
Side of hemiplegia (%)					0.571
Left	25(44.6)	11(40.7)	14(48.3)	
Right	31(55.4)	16(59.3)	15(51.7)	
Complication (%)					0.568
Neglect	5(8.9)	3(11.1)	2(6.9)		
Dysphasia	9(16.1)	3(11.1)	6(20.7)
Nil	42(75.0)	21(77.8)	21(72.4)
Site of lesion (%)					0.635
Cortex	11(19.6)	6(22.2)	5(17.2)		
Corona radiata	7(12.5)	4(14.8)	3(10.3)		
Internal capsule	8(14.3)	5(18.5)	3(10.3)		
Putamen	1(1.8)	1(3.7)	0		
Thalamus	6(10.7)	2(7.4)	4(13.8)		
Other	23(41.1)	9(33.3)	14(48.3)		
Follow-up physiotherapy (%)					0.151
Ambulatory day hospital	38(67.9)	21(77.8)	17(58.6)		
Domiciliary physiotherapy	3(5.4)	2(7.4)	1(3.5)		
Other	2(3.6)	0	2(6.9)		
Nil	13(23.2)	4(14.8)	9(31.0)		
Type of carer (%)					0.572
No	15(26.8)	10(37.0)	5(17.2)		
Spouse	11(19.6)	5(18.5)	6(20.7)		
Maid	6(10.7)	1(3.7)	5(17.2)		
Children	21(37.5)	10(37)	11(37.9)		
Other	3(5.4)	1(3.7)	2(6.9)		
Experience in smart device					0.598
<0.5 year	8(14.3)	3(11.1)	5(17.2)		
1–2 years	8(14.3)	5(18.5)	3(10.3)		
>2 years	40(71.4)	19(70.4)	21(72.4)		
MFAC	4.0(1.4)	4.3(1.0)	3.8(1.7)	0.173	
MBI	58.0(26.3)	65.1(20.7)	51.4(29.4)	0.048*	

*Notes*: Exp: experimental group; Con: control group; difference between groups by independent t-test or Chi-Square test. *p<0.05.

There were no between-group difference in baseline adherence (p=0.214), 1 month adherence (p=0.072) but significant difference in 3 months adherence (p=0.021) (Table 3).

**Table 3.** Exercise adherence and self-efficacy for exercise of experience and control groups.

	Groups		Between-group	Groups		Between-group	Groups		Between-group
	Baseline			1-Month			3-Month		
	Exp (n=27)	Con (n=29)	p-value	Exp (n=27)	Con (n=29)	p-value	Exp (n=27)	Con (n=29)	p-value
Adherence
Mean (SD)	74.1	64.1	0.214	73.7	58.6	0.072	75.6	55.2	0.021*
	(24.4)	(34.0)		(21.5)	(37.3)		(26.2)	(35.8)
SEE
Mean (SD)	52.5	48.1	0.307	58.4	43.3	0.001*	62.2	45.6	<0.000*
	(15.2)	(16.7)		(11.7)	(20.0)		(10.7)	(18.8)

*Notes*: Exp: experimental group; Con: control group; difference between groups of Adherence by Chi-Square test; difference between groups of SEE by independent t-test.

* p<0.05.

There were significant between-group difference in 1 month change of SEE (p=0.001) and 3 month change in SEE (p<0.000). There were no between-group difference in 1 month change of MFAC but significant between-group difference in 3 month change of MFAC (p=0.036). There were no between-group difference in 1 month change of MBI (p=0.474) and 3 month change in MBI (p=0.808) (Table 4).

**Table 4.** Functional outcomes of experience and control groups.

	Groups						Within-groups		Between-group	Within-groups		Between-group
	Baseline		1-month		3-month		1-month gain (1-month minus baseline)			3-month gain (3-month minus baseline)		
	Exp (n=27)	Con (n=29)	Exp (n=27)	Con (n=29)	Exp (n=27)	Con (n=29)	Exp (n=27)	Con (n=29)	p-value	Exp (n=27)	Con (n=29)	p-value
MFAC
Mean (SD)	4.3	3.8	5.1	4.3	6.0	4.8	0.9	0.5		1.7	1.0	
	(1.0)	(4.3)	(1.1)	(1.6)	(1.2)	(1.8)	(0.9)	(0.6)		(1.2)	(1.0)	
p-value							<0.000*	<0.000*	0.124	<0.000*	<0.000*	0.036*
MBI
Mean (SD)	65.1	51.4	79.0	63.2	85.4	70.8	13.9	11.9		20.9	19.4	
	(20.7)	(29.4)	(15.6)	(30.0)	(17.3)	(29.0)	(12.0)	(8.4)		(13.9)	(13.1)	
p-value							<0.000*	<0.000*	0.474	<0.000*	<0.000*	0.808

*Notes*: Exp: experimental group; Con: control group; difference within groups by pair *t*-test; difference between groups by independent t-test.

* p< 0.05.

## Discussion

The main finding of the study was that mobile video-guided home exercise program was superior to standard paper-based home exercise programs in terms of exercise adherence, SEE and mobility gain but not basic ADL gain for patients recovering from stroke. This finding contrasted to the study of Emmerson *et al.* which showed that home exercise programs filmed on an electronic tablet was not superior to standard paper-based home exercise programs in terms of adherence, motor function, or satisfaction for patients recovering from stroke.^12^ There are several possible explanations for these finding, the adherence of home exercise may depend on the type of exercise and interest of patients. It was suggested that mobility decline is an essential concern in chronic stroke patients.^42^ Emmerson’s study^12^ only provided upper limb exercises that patients may have less interest to participate than the exercises in this study which consisted for arm, leg, trunk and mobility. We also found that there was a close relationship between exercise adherence and SEE. During the 3 months follow-up period, the SEE slightly increased (from 52.5 to 62.2 in video group) with the exercise adherence (from 74.1% to 75.6%) in experimental group; whereas the SEE was slightly decreased (from 48.1 to 45.6) with exercise adherence (from 64.1% to 55.2%) in control group (Table 3). The present results echoed with the theory of self-efficacy, the stronger the individual’s self-efficacy, the more likely it is that people will initiate and persist with a given activity^16,45^ that SEE was positive proportion to exercise adherence in both groups. The study also found that video-guided home exercise program could improve exercise adherence and SEE even without any regular encouragement, or automated reminder as in previous studies by Lambert *et al.*^13^ and Emmerson *et al.*^12^ The higher mobility gain of patients in experimental group than control group could be explained by adherence of home exercise program that has positive correlation with physical function and physical performance of patients.^28,29^ No difference in MBI gain in experimental group when compared to control group means the video-guided exercise program improves mobility or ambulation (as measured by MFAC) but its effects could not be translated to improvement of ADL (as measured by MBI). This could be explained by specificity principle^46^ that in training the home exercise program in this study was motor control and mobility orientated but not ADL orientated.

The limitation of the study included information bias introduced by outcome collection by telephone interview; selection bias to Chinese population since all the videos and pamphlets were in Chinese; and confounding bias by confounding variables such as, by chance, the experimental group had significant higher baseline MBI than that of control group. However, the effects of the confounding bias have been minimized by randomization and statistical analysis. The intervention fidelity monitoring is another limitation of the study; a pre-discharge training session was used to minimize the limitation. Further research studies with large sample size would allow comparing the most effective groups of the video-guided home exercise. In addition, as mentioned in the introduction session, it seems that adherence was affected by both self-efficacy and delivery mode of training program. The relationship between self-efficacy and delivery mode of training program such as video-guided element is still unclear and needs further study.

## Conclusion

The use of mobile video-guided home exercise program was superior to standard paper-based home exercise program in exercise adherence, SEE and mobility gain but not basic ADL gain for patients recovering from stroke.

## Conflict of Interest

The authors declared no potential conflicts of interest with respect to the research, authorship, and/or publication of this paper.

## Funding/Support

The authors received no financial support for the research, authorship, and/or publication of this paper.

## Author Contributions

(1) Bryan Ping Ho Chung: Specifying the question, designing the study, analyzing the data, collecting and handling data, interpreting the data, writing — lead authorship.
(2) Wendy Kam Ha Chiang: Specifying the question, designing the study, collecting and handling data, interpreting the data, writing — contributing significant text.
(3) Herman Lau: Specifying the question, designing the study.
(4) Titanic Fuk On Lau: Specifying the question, translating a protocol into practice.
(5) Charles Wai Kin Lai: Translation of protocol into practice.
(6) Claudia Sin Yi Sit: Identifying data needed, identifying relevant references.
(7) Ka Yan Chan: Translation of protocol into practice.
(8) Chau Yee Yeung: Translation of protocol into practice.
(9) Tak Man Lo: Translation of protocol into practice.
(10) Elsie Hui: Translation of protocol into practice, reading, editing, checking.
(11) Jenny Shun Wah Lee: Translation of protocol into practice, reading, editing, checking.
